# Neutrophil kinetics in health and disease

**DOI:** 10.1016/j.it.2010.05.006

**Published:** 2010-08

**Authors:** Charlotte Summers, Sara M. Rankin, Alison M. Condliffe, Nanak Singh, A. Michael Peters, Edwin R. Chilvers

**Affiliations:** 1 Department of Medicine, University of Cambridge School of Medicine, UK; 2 Leukocyte Biology Section, National Heart and Lung Institute, Faculty of Medicine, Imperial College London, UK; 3 Division of Clinical and Laboratory Investigation, Brighton and Sussex Medical School, UK

## Abstract

Neutrophils play a key role in the elimination of pathogens. They are remarkably short-lived with a circulating half life of 6–8 h and hence are produced at a rate of 5 × 10^10^–10 × 10^10^ cells/day. Tight regulation of these cells is vital because they have significant histotoxic capacity and are widely implicated in tissue injury. This review outlines our current understanding of how neutrophils are released from the bone marrow; in particular, the role of the CXC chemokine receptor 4/stromal-derived factor 1 axis, the relative size and role of the freely circulating and marginated (i.e. slowly transiting) pools within the vascular compartment, and the events that result in the uptake and removal of circulating neutrophils. We also review current understanding of how systemic stress and inflammation affect this finely balanced system.

## Neutrophil homeostasis

Neutrophils are the most abundant circulating leukocyte in humans and play a fundamental role in the innate immune response. This is best exemplified by patients with neutropenia, chronic granulomatous disease or leukocyte adhesion deficiency syndrome, who are particularly prone to bacterial and fungal infection. Neutrophils are recruited rapidly to sites of inflammation, where their primary role is to kill invading bacteria and certain fungal species through phagocytosis by release of preformed granular enzymes and proteins, and by the production of a range of oxygen species. However, the highly destructive capacity of these cells also raises the potential for neutrophils to damage healthy tissues, which occurs in many inflammatory diseases such as acute respiratory distress syndrome, inflammatory bowel disease, and rheumatoid arthritis. Neutrophil abundance, coupled with their brief (6–8 h) circulating half life, mandates a basal rate of production by the bone marrow of 5 × 10^10^–10 × 10^10^ neutrophils/day; the advantage to the host of this rapid turnover is uncertain. Neutrophil homeostasis is maintained by a fine balance between granulopoiesis, bone marrow storage and release, intravascular margination, clearance and destruction. Margination refers to the prolonged transit of neutrophils through specific organs, which results in discrete intravascular (marginated) pools; these can be found within the spleen, liver, bone marrow and, more controversially, the lung. This review outlines our current understanding of the mechanisms that govern these processes.

## Neutrophil production in bone marrow

Neutrophils are produced within haematopoietic cords interspersed within the venous sinuses of the bone marrow. Granulocyte differentiation is regulated by the coordinated expression of key myeloid transcription factors, with granulocytes and macrophages differentiating from a common committed progenitor cell. Transcriptional profiling studies suggest that macrophages represent the default myeloid cell, and that granulocytes arise through the selective expression of a subset of transcription factors (e.g. Egr1, HoxB7 and STAT3), proteins (e.g. S100A8, S100A9 and neutrophil elastase) and receptors (e.g. for N-formyl methionyl-leucyl-phenylalanine and granulocyte-macrophage colony-stimulating factor (GM-CSF)) [1]. Recent data have indicated for that the small GTPase Rac2 also contributes to generating the myeloid lineage in haematopoietic cells [2]. The area of myelopoiesis and the transcription factors involved has been reviewed extensively elsewhere [3,4].

The neutrophil population in the bone marrow can be subdivided into three pools: the stem cell pool, the mitotic pool and the post-mitotic pool. The stem cell pool consists of undifferentiated haematopoietic stem cells (HSCs), whereas the mitotic pool refers to committed granulocytic progenitor cells that are undergoing proliferation and differentiation. Finally, fully differentiated mature neutrophils make up the post-mitotic pool, which forms the bone marrow reserve, available for release. Studies that have used cells labelled with ^32^P or ^3^H have shown that, in humans, the transit time through the post-mitotic pool is 4–6 days [5,6]. Likewise, the bone marrow reserve of granulocytes in humans has been estimated at 6 × 10^11^ cells; given this, and the assumption of a 5-day lag between DNA labelling of cells in the marrow and their appearance in blood, the daily turnover of granulocytes through the blood should be 1.7 × 10^9^ cells/kg, which accords well with experimental observations [7–9].

The principal regulator of physiological granulopoiesis is granulocyte colony stimulating factor (G-CSF) whose effects include commitment of progenitor cells to the myeloid lineage [10], proliferation of granulocytic precursors, reduction of transit time through the granulocytic compartment [11], and release of mature cells from the bone marrow. G-CSF exerts its effects through the G-CSF receptor, which is a member of the class I cytokine receptor family. Mice that lack the G-CSF receptor [12,13] and humans who express a dominant negative receptor mutation [14,15] are profoundly neutropenic. Interleukin (IL)-6, GM-CSF and IL-3 also stimulate granulopoiesis *in vivo* [16–18], but in all three cases, single knock-out mice exhibit normal basal levels of granulopoiesis [19–21], which suggests significant redundancy or reserve. This conclusion is strengthened by a recent study which has demonstrated that, although basal granulopoiesis is impaired in G-CSF and GM-CSF double knockout mice, IL-6 stimulates increased neutrophil production in response to lipopolysaccharide (LPS) [22].

## Neutrophil release from bone marrow

To exit the bone marrow, mature neutrophils must migrate across the sinusoidal endothelium that separates the haematopoietic compartment from the circulation. Neutrophils migrate across the bone marrow endothelium through tight-fitting pores by a unique process of transcellular migration, and pass through the cell body of the endothelium, rather than at cell–cell junctions [23–25]. Neutrophils maintain G-CSF receptors at high levels on their surface from early in their development [26]; CXC chemokine receptor 4 (CXCR4), a G-protein coupled receptor, is also expressed at low levels on the cell surface of mature neutrophils. The major ligand for CXCR4 is stromal-derived factor 1 (SDF-1), a CXC chemokine that is produced constitutively by bone marrow stromal cells. The interaction between CXCR4 and SDF-1 retains neutrophils within the marrow environment, as demonstrated by the rare autosomal dominant disorder WHIM (warts, hypogammaglobulinaemia, infection and myelokathesis [27]). WHIM patients are profoundly neutropenic despite having increased numbers of neutrophils in the bone marrow. These patients have a mutation in *CXCR4,* which is proposed to be responsible for impaired neutrophil release from the bone marrow, by enhancing sensitivity to SDF-1 and promoting retention [28,29]. The role of CXCR4-SDF-1 interaction in regulating neutrophil egress from the bone marrow is further supported by the finding that *Cxcr4* deletion in murine myeloid cells results in increased neutrophil release [30], and by the observation that treatment with a CXCR4 antagonist or blocking antibodies leads to rapid mobilization of neutrophils from both human and mouse bone marrow [31–33].

The CXCR4-SDF-1 axis is also important for the retention of HSCs and as a consequence disruption of CXCR4-SDF-1 interaction offers the potential to enhance the mobilization of HSCs for therapeutic harvesting [34,35]. Co-administration of G-CSF with a CXCR4 antagonist results in synergistic HSC release. One mechanism by which G-CSF exerts its multiple effects on neutrophil homeostasis is by inhibiting the CXCR4-SDF-1 axis. Treatment of mice with G-CSF decreases stromal cell SDF-1 production, which correlates with an increase in neutrophil release [36]; in a further study, G-CSF reduced CXCR4 surface expression specifically on myeloid cells [37]. Experiments in transgenic mice that express various G-CSF receptor mutations have shown a strong correlation between the magnitude of neutrophil mobilization and the reduction in SDF-1 protein expression by bone marrow [38].

The α_4_ intergrin very late antigen-4 (VLA-4) is expressed by neutrophils and might mediate their adhesion to bone marrow stromal cells and endothelium, which both express the ligand for VLA-4, vascular cell adhesion molecule-1 (VCAM-1). The possibility of cross-talk between the CXCR4-SDF-1 axis and the VLA-4-VCAM-1 interaction has been proposed as important for the retention and release of neutrophils from the bone marrow under homeostatic conditions, and furthermore, signalling through CXCR4 might affect neutrophil release by modulating the VLA-4-VCAM-1 adhesive interaction [39]. Expression of VLA-4 is downregulated during neutrophil maturation in the bone marrow, and α_4_ blockade increases mobilization of neutrophils from the bone marrow. Neutralization of CXCR4 and VCAM-1 results in a significant increase in circulating neutrophils, which suggests cross-talk between the CXCR4-SDF-1 and VLA-4-VCAM-1 axes.

## Circulating and marginated pools of granulocytes

**Figure 1:**
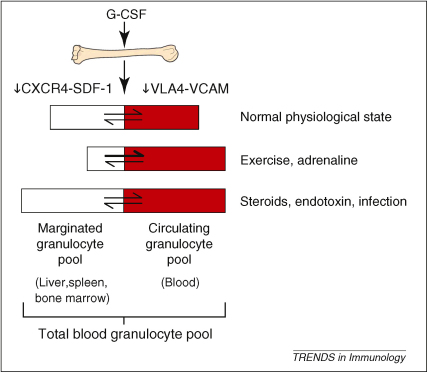
**Factors that affect the size of the marginated and circulating granulocyte pools.** G-CSF is the principal regulator of granulopoiesis and affects the commitment of progenitor cells to the myeloid lineage, proliferation of granulocytic precursors, as well as reducing transit time through the granulocyte compartment and stimulating the release of mature cells from the bone marrow. Neutrophil release from the bone marrow into the total blood granulocyte pool reflects loss of interaction between CXCR4 and SDF-1, and between VLA-4 and VCAM-1. Neutrophils within the total blood pool then enter either the marginated or freely circulating granulocyte pool that are of similar size in humans. A number of factors including exercise, drugs and infection affect the relative size of these two pools, as indicated.

Almost 50% of ^32^P-labelled autologous granulocytes injected into healthy volunteers disappear from the circulation after infusion [40]. However, the number of neutrophils that remain in the circulation can be increased by the addition of adrenaline to the infusion bag [41]. It is therefore considered that a proportion of the granulocytes that exit the circulation could be mobilized back into this freely circulating pool; this recoverable portion of granulocytes is termed the marginated pool. It has been estimated that the total blood granulocyte pool is 65 × 10^7^ cells/kg, with 49% of cells residing in the circulating pool and the remaining 51% in the marginated pool [7]. Prednisolone increases the size of both the circulating and marginated pools, while exercise and adrenaline cause a shift of cells from the marginated to the circulating pool. Bacterial endotoxin increases the size of both the total blood granulocyte pool by increasing bone marrow release, as well as the proportion of cells within the marginated pool. These pioneering studies have underpinned the modern concept of circulating and marginated intravascular granulocyte pools (Figure 1).

## The marginated neutrophil pool

The size of an individual marginated pool is the product of the mean intravascular transit time through the organ (i.e. the mean time taken for neutrophils to pass through the capillary bed) and its blood flow. Using radiolabelled neutrophils and a range of analytical techniques, mean neutrophil intravascular transit time has been measured for the liver (∼2 min [42]), spleen (∼10 min [42]) and bone marrow (∼10 min [43]).

The size of the pulmonary marginated granulocyte pool is more controversial. A large body of data suggest that the lung is the predominant site of physiological neutrophil margination [44], but this has been called into question by data obtained using leukocyte scintigraphy, which demonstrate that the lungs of healthy humans are only modestly engaged in physiological neutrophil pooling [45]. The lungs receive the entire cardiac output, therefore, the size of the pulmonary granulocyte pool is determined predominantly by the mean pulmonary transit time. There has been little consensus regarding this variable, and a plethora of experimental methodologies have been applied in attempts to produce definitive results, with limited success (reviewed previously [45]).

The biodistribution of neutrophils is also determined by their maturation and activation status; this might be relevant to the controversy outlined above, because techniques used to purify neutrophils for such studies might inadvertently activate them. This is highlighted by leukocyte scintigraphy in subjects injected with granulocytes purified with Percoll-saline density gradients and labelled with ^111^In, which show marked retention in the lungs and slow accumulation in the liver and spleen [45]. Mature peripheral blood neutrophils have been shown to localize to the liver, bone marrow and, to a lesser extent, the spleen; younger marrow-derived cells home back mainly to the bone marrow, while inflammatory peritoneal neutrophils localize predominantly to the liver, but also to the lungs [46]. These latter post-migratory neutrophils are highly activated and their biodistribution might be non-physiological. However, this might help to predict the behaviour of circulating neutrophils in systemic inflammatory disease.

## Neutrophil uptake and removal by the liver, spleen and bone marrow

After injection into healthy individuals, neutrophils leave the vascular compartment with a time course that declines exponentially and a half life of about 7 h, and undergo destruction in the reticuloendothelial system [47]. The mechanisms of physiological neutrophil destruction *in vivo* are poorly defined.

**Figure 2:**
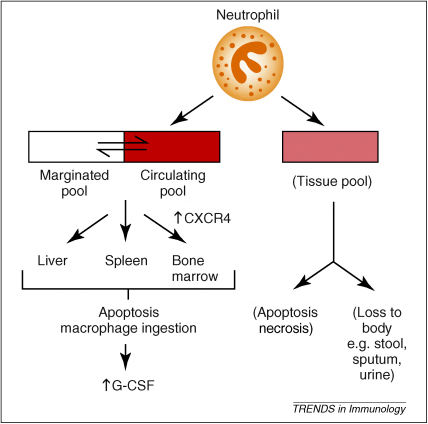
**Physiological fate of granulocytes within the blood pool.** The absence of infection or injury (which results in the targeted migration of neutrophils into the inflamed tissues), neutrophils exit the total blood granulocyte pool and can be found in the liver, spleen and bone marrow in approximately equal proportions. Neutrophil apoptosis within these tissues results in macrophage recognition and ingestion that leads to G-CSF generation, which in turn stimulates granulopoiesis. Neutrophils within inflamed tissue undergo apoptotic or necrotic cell death or are lost to the body following trans-epithelial migration.

We and others [48–50] have identified in the mouse a senescent neutrophil phenotype that has increased surface expression of CXCR4, which develops just before apoptosis. It is proposed that under physiological conditions, upregulation of CXCR4 on circulating neutrophils supports homing to the bone marrow, where these cells undergo apoptosis and are phagocytosed by stromal macrophages. This in turn stimulates G-CSF production, which provides the required homeostatic link between clearance and production/release (Figure 2). Homing of senescent neutrophils to the bone marrow is inhibited by pertussis toxin, which supports a role for a G-protein receptor such as CXCR4 in this process [45]. This model implies a dual role for CXCR4 in neutrophil homeostasis, where it both retains neutrophils in the marrow until they reach maturity, and acts as a signal to home senescent cells to the marrow for destruction. The relevance of this signalling pathway in humans is uncertain.

*Cxcr4* deletion is embryonically lethal [51], however, data from a myeloid-specific *Cxcr4* mouse knockout suggest that CXCR4 is not the only regulator of neutrophil clearance from the circulation [52]. Mice that lack myeloid CXCR4 exhibit circulating neutrophilia, with premature release of neutrophils from the bone marrow. Adoptive transfer of knockout cells to wild type animals has demonstrated decreased homing to the bone marrow, which is consistent with previous findings [49]. However, neutrophils that lack CXCR4 have a circulating half life that is not significantly different from that of wild type cells; this suggests that while CXCR4 might promote the homing of senescent neutrophils to the bone marrow, other (as yet unidentified) factors and/or mechanisms are also important in regulating this process. This resonates with the observations that neutrophil disappearance from the blood (unlike erythrocyte loss) follows a time course that fits to a mono-exponential curve, which is a pattern that is not consistent with exclusively senescence-based destruction.

A role for the liver in regulating circulating neutrophil numbers has also been proposed. The liver is thought to offer a clearance pathway in which neutrophil phagocytosis is undertaken by hepatic Kupffer cells after P-selectin-mediated hepatic sequestration [53]. Selective upregulation of P-selectin in response to low-dose LPS has been found within the liver (but not the lungs or spleen), and phagocytosis of circulating apoptotic neutrophils is almost exclusively limited to the liver, with cells being phagocytosed by Kupffer cells through surface phosphatidylserine interactions.

## Effects of systemic stress and inflammation on neutrophil kinetics

Stress and systemic inflammation are associated with circulating neutrophilia and multiple inflammatory mediators including leukotriene B4, complement component C5a, IL-8 and tumour necrosis factor-α (TNFα) have been shown to induce neutrophilia when injected into experimental animals. It has been suggested that mobilization of neutrophils from a marginated pool within the bone marrow sinusoids explains this increase [54]. However, more recent data have suggested that cells are mobilized from the haematopoietic compartment in response to concentration gradients across the sinus wall of bone marrow sinusoids, which are generated by the production of mediators such as macrophage inflammatory protein-2, G-CSF and CXCL1 (KC) at inflammatory sites [25]. The precise mechanism by which inflammation leads to circulating neutrophilia is incompletely understood. However, acute mobilization of neutrophils from the bone marrow might require the coordinated, yet distinct, actions of G-CSF and CXC chemokines, with G-CSF disrupting the CXCR4-SDF-1 retention mechanism and CXC chemokines stimulating neutrophil chemotaxis across the bone marrow endothelium [55].

A role for SDF-1 in attracting a second influx of neutrophils into the lungs in acute lung injury has also been suggested [56]. SDF-1 expression (both protein and mRNA) has been found to be increased in the pulmonary epithelium in patients with histologically proven acute lung injury and mice with LPS-induced pneumonitis. Additionally, upregulation of CXCR4 expression has been noted on the cell surface of the neutrophils that have migrated into the lung, and neutrophil migration is ameliorated by the use of an SDF-1 blocking antibody.

The histotoxic potential of neutrophils dictates the need for effective processes to prevent inappropriate tissue-specific accumulation and activation. The key mechanism for this is priming, which dictates that neutrophil activation is a two-step process that requires an initial exposure to mediators such as cytokines. These factors can be divided into early-phase cytokines such as TNFα, IL-1β, and pathogen associated molecular patterns (PAMPs) such as endotoxin, or late-phase chemoattractants and growth factors including IL-8, LTB4 and GM-CSF. Priming can also be induced by the interaction of neutrophils with activated endothelial surfaces (previously reviewed [57]). Maximal neutrophil degranulation and activation of the NADPH oxidase occurs only in cells that have been primed before activation [58]. Crucially, priming is also an absolute requirement for neutrophil-mediated tissue injury, and affects neutrophil cytoskeletal organization to induce shape change and reduce deformability [59]; this causes stiffening of the neutrophils, which makes them more prone to retention in capillary beds. Neutrophil priming, including shape change, has been shown to be reversible *in vitro* [60], however, there are limited data about the effects of priming on neutrophil kinetics *in vivo*.

**Figure 3:**
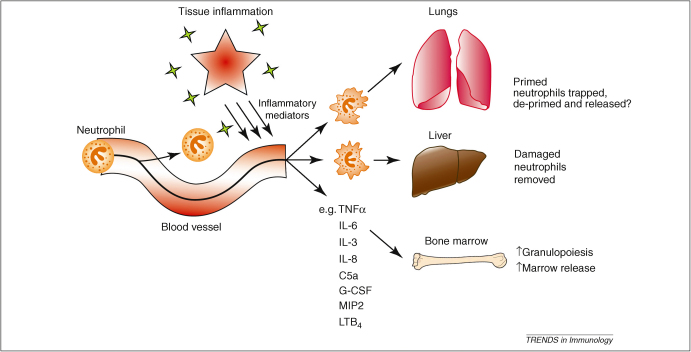
**Effects of local and systemic inflammation on neutrophil kinetics.** Tissue inflammation results in the release of multiple inflammatory mediators and subsequent neutrophil priming. Priming results in a marked change in neutrophil shape and rheology that leads to retention within the capillary microvascular bed of the lung. The liver is thought to play a particularly important role in recognizing and removing damaged neutrophils. Circulating inflammatory markers stimulate granulopoiesis and rapid release of preformed mature neutrophils from the bone marrow.

Up to 15% of the cardiac output can pass through an inflamed site (as observed in patients with active inflammatory bowel disease), therefore, all neutrophils are expected to be exposed to the priming stimulus within minutes. However, *in vivo* studies in inflammatory disorders have found a maximum of 60% of circulating neutrophils in a primed state [61,62]. Assuming that all neutrophils that emerge from an inflammatory site are primed, and that the life span of a primed neutrophil is no shorter than an unprimed one (6–8 h), de-priming seems mathematically justified. For example, during inflammation, the rate of priming is equal to the sum of the rate of de-priming and the rate of destruction; consequently, if no de-priming occurs, such studies should demonstrate that at least 97% of circulating neutrophils are in the primed state, but this has not been the case, suggesting that de-priming might occur *in vivo*. The lungs receive the entire cardiac output, and thus have the potential to protect the systemic circulation, therefore, they offer a possible site of neutrophil de-priming where primed cells are retained until they de-prime and can be returned to the systemic circulation in a safe, quiescent state (Figure 3).

If the lungs afford a site for physiological neutrophil de-priming, it should be possible to measure a gradient of primed cells across the pulmonary vascular bed. The trans-pulmonary gradient of H_2_O_2_ production (a marker of priming/activation status) by zymosan-activated neutrophils has been examined in septic patients without lung infiltrates, in patients with lung injury, and control patients who are undergoing elective surgery [63]. Septic patients have higher H_2_O_2_ in mixed venous blood (blood yet to enter the pulmonary circulation) compared with arterial blood (blood that has left the pulmonary circulation), which is consistent with the idea that neutrophils primed in the periphery are sequestered and de-primed within the lungs. Patients with lung injury, however, have higher levels of H_2_O_2_ in arterial blood, which suggests that the de-priming mechanism fails and the lungs themselves become a site of neutrophil priming. Control patients have no measurable gradient of H_2_O_2_ production between venous and arterial blood samples. Further evidence from an animal model also implicates the lung as a potential site of *in vivo* de-priming. In rats, neutrophil priming has been compared between blood entering and exiting the lungs [64]; 4 h after experimentally generated pneumonia, a higher proportion of primed neutrophils enter the lungs than leave, as quantified by the presence of F-actin rims that are visible within primed cells. This demonstrates that the lungs do sequester primed neutrophils and therefore might protect the systemic circulation from the potentially damaging effects of primed cells.

## Conclusions

The crucial role of neutrophils in innate immunity, coupled with their proclivity to cause tissue injury, mandate that their formation, mobilization and clearance are tightly controlled. G-CSF regulates myelopoiesis, while the cytokine responsive CXCR4-SDF-1 signalling axis acts as the principal regulator of bone marrow neutrophil retention/release. The factors that are responsible for the removal of neutrophils from the circulation are unclear, but upregulation of CXCR4 might contribute to homing of neutrophils to the bone marrow for disposal. The lungs are now thought to play little part in physiological neutrophil sequestration, but could have an important protective role by filtering primed neutrophils from the circulation and thus facilitating de-priming. The potentially protective role of the lungs, and whether this is a passive or active process, requires further investigation, but it could lead to novel therapeutic strategies for inflammatory conditions. Further work is also required on the potential of PAMPS and Damage-associated molecular pattern molecules (DAMPs) to induce neutrophil priming and modulate neutrophil kinetics. Although we have gained new insights into the mechanisms of neutrophil release from the bone marrow, the molecular mechanisms that regulate their re-uptake in the liver, spleen and bone marrow are less certain and warrant further investigation.
